# Individual heterogeneity in black brant survival and recruitment with implications for harvest dynamics

**DOI:** 10.1002/ece3.767

**Published:** 2013-09-19

**Authors:** Mark S Lindberg, James S Sedinger, Jean-Dominique Lebreton

**Affiliations:** 1 Institute of Arctic Biology and Department of Biology and Wildlife, University of Alaska FairbanksFairbanks, Alaska, 99775; 2 Department of Natural Resources and Environmental Science, University of NevadaReno, Nevada, 89557; 3 C.E.F.E., UMR 5175, C.N.R.S.Route de Mende, 34293, Montpellier Cedex 5, France

**Keywords:** Adaptive management, frailty, growth rate, harvest, waterfowl

## Abstract

We examined individual heterogeneity in survival and recruitment of female Pacific black brant (*Branta bernicla nigricans*) using frailty models adapted to a capture–mark–recapture context. Our main objectives were (1) to quantify levels of heterogeneity and examine factors affecting heterogeneity, and (2) model the effects of individual heterogeneity on harvest dynamics through matrix models. We used 24 years of data on brant marked and recaptured at the Tutakoke River colony, AK. Multievent models were fit as hidden Markov chain using program E-SURGE with an adequate overdispersion coefficient. Annual survival of individuals marked as goslings was heterogeneous among individuals and year specific with about 0.23 difference in survival between “high” (0.73)- and “low” (0.50)-quality individuals at average survival probability. Adult survival (0.85 ± 0.004) was homogeneous and higher than survival of both groups of juveniles. The annual recruitment probability was heterogeneous for brant >1-year-old; 0.56 (±0.21) and 0.31 (±0.03) for high- and low-quality individuals, respectively. Assuming equal clutch sizes for high- and low-quality individuals and that 80% of offspring were in the same quality class as the breeding female resulted in reproductive values about twice as high for high-quality individuals than low-quality individual for a given class of individuals producing differential contributions to population growth among groups. Differences in reproductive values greatly increased when we assumed high-quality individuals had larger clutch sizes. When we assumed that 50% of offspring were in the same quality class as their mothers and clutches were equal, differences in reproductive values between quality classes were greatly reduced or eliminated (breeders [BRs]). We considered several harvest scenarios using the assumption that 80% of offspring were in the same quality class as their mothers. The amount of compensation for harvest mortality declined as the proportion of high-quality individuals in the harvest increased, as differences in clutch sizes between groups decreased and as the proportion of BRs in the harvest increased. *Synthesis and applications*. Harvest at the same proportional level of the overall population can result in variable responses in population growth rate when heterogeneity is present in a population. λ was <1.0 under every scenario when harvest rates were >10%, and heterogeneity caused as much as +2% difference in growth rates at the highest levels of proportional harvest for low-quality individuals and the greatest differences in qualities between classes of individuals, a critical difference for a population with λ near 1.0 such as the brant. We observed less response in overall survival in the presence of heterogeneity because we did not observe heterogeneity in the annual survival of BRs. This analysis provides a comprehensive view of overall compensation at the population level and also constitutes the first example of a survival-recruitment model with heterogeneity. Individual heterogeneity should be more explicitly considered in harvest management of vertebrates.

## Introduction

The harvest of wildlife and fisheries populations has been the subject of considerable debate for almost a century (Baranov 1918; Beverton and Holt 1957). Difficulty with monitoring fish stocks and unexpected changes in harvest encouraged the use of modeling (e.g., Getz and Haight 1989) and the development of theory to guide harvest management, which was then applied to a number of North American waterfowl populations (Anderson and Burnham 1976). More recently, the issue of harvest was examined as a conservation problem (Reynolds et al. 2001), and symmetrically incidental exploitation (e.g., diffuse mortality induced by human activities in an otherwise protected species) was considered as an exploitation problem (Lebreton 2005). Whatever the context, a central question in harvest dynamics is that of compensation; does harvest, as a specific cause of mortality, add its effects totally independently of natural mortality or is the effect partially or totally compensated?

Harvest management of most waterfowl, particularly in North America, is currently guided by the assumption that harvest mortality is at least partially additive (Johnson et al. 1993; Conn and Kendall 2004), and if compensation occurs, it is primarily through density dependence in survival probability and reproduction (Anderson and Burnham 1976; Nichols et al. 1995). Harvest mortality may be compensated through density-dependent increases in survival or reproduction postharvest, such that harvest mortality may have no effect on overall survival or growth rate of the population. The evidence for additive or compensatory harvest mortality is mixed (Nichols et al. 1984; Rexstad 1992; Smith and Reynolds 1992; Gauthier et al. 2001; Williams et al. 2002; Sedinger et al. 2007; Sedinger and Herzog 2012) and is least compensated in populations with higher inherent survival probability. Evidence for density-dependent regulation of survival and reproduction in waterfowl is inconsistent (Johnson et al. 1992; Anderson et al. 1997; Sedinger et al. 1998; Viljugrein et al. 2005) and may be too weak for compensation to occur (Lebreton 2005). Moreover, detecting density dependence (Lebreton 2009) or a negative correlation between natural mortality and harvest (Schaub and Lebreton 2004) is the subject of notable statistical difficulties (Otis and White 2004). As a consequence, some retrospective analyses may overestimate the prevalence of density dependence (Shenk et al. 1998; Lebreton 2005). Furthermore, distinguishing between the effects of harvest and density on abundance is difficult because harvest regulations are typically liberal when abundance is high and conservative when abundance is low (Smith and Reynolds 1992; Sedinger and Rexstad 1994; Sedinger and Herzog 2012).

Because of the continuing debate about the role of density dependence in compensation, alternative functional forms of the relationship between harvest and population change may be required to fully represent these dynamics (Runge and Johnson 2002; Conn and Kendall 2004). We examined how heterogeneity or individual variation in survival and recruitment of Pacific black brant (*Branta bernicla nigricans*; hereafter brant) may provide an alternate explanation for the relationship between harvest and population dynamics. Others (Johnson et al. 1984, 1988; Lebreton 2005) previously demonstrated through statistical and population models that individual heterogeneity in survival and reproduction can lead to compensation even in the absence of density dependence, and we further these findings using data on a specific population and new modeling tools.

The consequences of harvest are linked to the expected contributions of the harvested individuals to future population growth, which are measured by reproductive value (MacArthur 1960; Kokko 2001). Harvest of an individual with high reproductive value has more effect on population dynamics than harvest of an individual of low reproductive value (Brooks and Lebreton 2001; Kokko 2001; Hauser et al. 2006). Disproportionate harvest risk of individuals in poor physiological condition (Greenwood et al. 1986; Dufour et al. 1993) is consistent with the hypothesis that heterogeneity in reproductive value provides some compensation for harvest mortality. Heterogeneity in reproductive value of individuals has been clearly linked to a number of characteristics (e.g., age, gender); however, unexplained sources of heterogeneity may have additional effects on reproductive value and harvest dynamics, and these sources of heterogeneity have created modeling challenges (Vaupel and Yashin 1985; Link et al. 2002).

In human health studies, individual heterogeneity is considered as a random variable with a continuous distribution in so-called “frailty” survival models (Vaupel 1990), and these models have also been successfully used in studies of free-ranging vertebrate (e.g., Cam et al. 2002). An alternative is to consider that demographic parameters are distributed according to a discrete distribution; the population is a mixture of several types of individuals differing in parameters such as survival probability. Pledger and Schwarz (2002) reviewed capture–recapture survival models with heterogeneity either continuous or based on finite mixtures, and they concluded that two-level mixtures (i.e., considering the population is composed of two types of individuals) often provided an adequate representation of individual heterogeneity (but see Dorazio and Royle 2003). We explored a novel application of finite mixture models to multiply demographic parameters.

Rich, longitudinal data sets are still needed to model individual heterogeneity. The long-term study of brant in western Alaska (e.g., Sedinger et al. 2008; Nicolai et al. 2012) presented an excellent opportunity for us to examine new methods for simultaneously modeling heterogeneity in capture, recruitment, and survival probability and explore how this variation may change through time. We use estimated levels of heterogeneity to explore implications of this heterogeneity for understanding effects of historical harvest rates for brant on population dynamics. Our main objectives were (1) to quantify levels of heterogeneity and examine factors affecting heterogeneity and (2) model the effects of individual heterogeneity on harvest dynamics through matrix models. We predicted that individual heterogeneity in survival and recruitment would exist in the brant population, and that heterogeneity would compensate for some harvest.

## Materials and Methods

### Study system and data collection

Data were collected at the Tutakoke River brant colony (61°159′N, 165°37′W) on the Yukon-Kuskokwim Delta, AK, which historically represented about 20% of the breeding population (Sedinger et al. 1993). Brant are long-distance migrants that winter along the Pacific coast of North America from Alaska to the Pacific coast of Mexico (Reed et al. 1998). Brant from the Tutakoke River colony nest in coastal tundra within 2 km of the Bering Sea coast. We captured individual brant by herding them into corral traps during the adult remigial molt in mid-late July each summer (Sedinger et al. 1997). We assigned individuals to one of three age classes upon initial capture based on plumage characteristics: goslings (HY, approximately 1 month old); 1-year-olds (SY,13 months old) and adults (ASY, ≥25 months old). We placed U.S. Geological Survey steel bands and uniquely engraved plastic bands on captured individuals; plastic bands could be read at a distance of >100 m with spotting scopes, which facilitated detection in subsequent years. We determined gender by cloacal examination. Goslings in nests associated with marked adults received uniquely numbered webtags while still in the nest (Sedinger and Flint 1991), which allowed us to determine ages of goslings (±1 day) when captured in banding drives. We weighed and measured all web-tagged goslings when captured during banding drives. We calculated a cohort mean mass adjusted for gender and age (days).

We restricted the analysis to females because (1) most males disperse to other breeding locations (Lindberg et al. 1998), reducing the number of known-age individuals available for study; and (2) brant maintain long-term pair bonds, so fates of males and females were not independent. We encountered marked individuals during the aforementioned banding drives and during nesting. However, few 1-year-olds were observed and none have ever been confirmed as breeders (BRs), encounters of 1-year-olds were therefore removed and we modeled initial survival over the first 2 years of life. At nesting, we visited 50, randomly located 50-m radius plots every 4 days during egg laying and at least on alternate days during the hatch period. We read plastic bands when females were flushed from their nests. Additionally, we attempted to check all nesting females on the colony for plastic bands by flushing them from their nests, beginning shortly after the end of egg laying. We detected about 79% of breeding females that were present on the colony annually (Sedinger et al. 2001).

### Multievent models

Analysis was based on female brant marked with individually coded tarsal tags and legbands at the Tutakoke River colony, AK from 1987 to 2009 (Sedinger et al. 1998). We examined individual heterogeneity of female brant based on CMR recruitment models (see e.g., Lebreton et al. 2003) incorporating individual heterogeneity in the various parameters of these models. The resulting model was a multievent model (Pradel 2005), which is a type of hidden Markov chain model (Choquet et al. 2009). We considered a suite of models both with and without individual heterogeneity and other sources of variation to estimate state (i.e., quality) of an individual and capture, apparent survival, and recruitment probability. We used an information-theoretic approach for model selection and to determine what sources of variation were supported (Burnham and Anderson 2002). When included, individual heterogeneity was modeled using a two-level mixture model with two hidden groups. This structure results in four states: 1 = BR group A, 2 = prebreeder (PB) group A, 3 = BR group B, 4 = PB group B. A shortcoming of this approach is that heterogeneity in capture probability is linked with heterogeneity in survival and recruitment, such that an individual in a specific survival or recruitment group has to be in the same group for detection probability. Separating these two types of heterogeneity would require four hidden groups or eight states. Such a model would be quite unstable and present severe identifiability problems and was not considered. The negative sampling correlations between parameters, however, will tend to exaggerate heterogeneity in the demographic parameters of interest, and we consider that issue in the Discussion.

As we are only sampling at a breeding colony, individuals are observed as a BR (only after second year) or PB (in their hatch year) at initial capture and as a BR in any subsequent recapture. However, we do not know to which heterogeneity state (A or B) an individual belongs because this is a hidden state. Therefore, the proportion of individuals in heterogeneity groups A and B have to be estimated within each breeding category. These initial-state probabilities come as extra parameters in multievent models when compared to usual multistate CMR models, in which the state of an individual at time of marking is known. A model with year-specific variation in initial state and 25 occasions would require 75 parameters because the 4th state is estimated as 1−the sum of the probability of the other three states.

Recapture probability (*p*_*i*_) was the probability that an individual alive and in the Tutakoke River population in year *i* was reencountered in that year. We considered models with effects of year and heterogeneity on *p*, with an additive and interactive relationship between these factors. With *p* and other parameters, we considered models with linear trends (logit scale) in heterogeneity to examine whether heterogeneity changed through years because the size of the brant colony was declining during the study period, which may have changed the amount of heterogeneity through time. Apparent survival probability (ф_*i*_), the probability that a brant alive and associated with the Tutakoke colony at year *i* survives and does not permanently emigrate between year *i* and *i* + 1, was modeled as a function of age, year, mass at capture (i.e., mean year cohort mass adjusted for age; Sedinger and Nicolai 2011), and heterogeneity. Juvenile and adult survival probabilities were modeled independently. Juvenile survival spans the 2 years from fledging to potential age of first breeding, since second year encountered were removed. We estimated survival for the first and second year of life assuming survival was equal for these two annual periods. Recruitment probability (*a*_*j*_), the probability that animal of age *j* starts to breed at that age (Pradel and Lebreton 1999), was modeled as a function of age from 2-years old up to 6-years old with and without the effects of heterogeneity.

We assessed fit of the model to the data using tests conducted in program U-CARE following Crespin et al. (2006) for recruitment models and Fletcher et al. (2012) for models with heterogeneity. By definition, heterogeneity in capture probability corresponds to a mix of individuals with low and high capturability. Compared with homogeneity, the data set will present an excess of runs of 0 and of runs of 1. This situation results in the simultaneous presence of various degrees of transience and trap happiness, respectively, detected by goodness-of-fit (GOF) components TEST3.SR and TEST2.CT, as shown by Fletcher et al. (2012). We expect all other components to be somewhat sensitive to heterogeneity in all parameters.

All models were run with random initial values repeated 10 times to protect against local minima. In all cases, the minimal value of the deviance was that obtained for the largest number of repeats, so we are confident that the deviances used in QAIC calculation corresponded to the global minimum. For a few of the most complex models, the deviance was obtained after 200 iterations and full convergence was not yet attained, implying a slight overestimation not bearing any consequence on QAIC-based model selection. This was always due to the slow optimization of boundary estimates (i.e., a probability estimated to be 1.0, has to converge in logit to “infinity,” represented by the value 15 in E-SURGE), and this convergence requires many iterations, without substantial changes to the deviance. Our checks with a few of these models showed that the difference in deviance was always <0.100, far from the difference in QAIC with the preferred models.

### Matrix models

Using estimates from the best multievent models and published estimates of fecundity (Sedinger et al. 1998; Nicolai and Sedinger 2012), we parameterized a matrix model with two population segments (A and B) in a gosling, prebreeding, or breeding state (six states total) using a postbirth pulse structure (Caswell 2001). Matrix models were run in ULM (Legendre and Clobert 1995) and MATLAB® (Mathworks Inc., Natick, MA). We analyzed the matrix to determine the reproductive value of various states and changes in growth rate (λ) under harvest scenarios that included different proportions of brant from the six states. We calculated reproductive values in the presence of heterogeneity with different levels of inheritance of the states where differences in “heritability” represented individuals producing different proportion of goslings in their quality state. We use the term inheritance rather than heritability, to acknowledge that both genetic and environmental (e.g., timing of reproduction, brood-rearing area) factors may affect quality of offspring. We also considered how reproductive values and λ changed with differences in clutch size among quality classes.

Under the assumptions that females produced 80% offspring of the same class, and in addition to heterogeneity in survival and recruitment, we evaluated the effects of harvest with different proportions of quality classes in the harvest, different clutch sizes between classes, and different proportions of goslings, PBs, or BRs in the harvest. Scenarios for harvest rates were developed based on estimates from Sedinger et al. (2007). Harvest effects were evaluated by comparing resulting λ to the per capita growth rate expected under a uniform harvest proportion (*h*) irrespective of reproductive value:


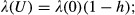


where λ(0) is the growth rate in the absence of harvest and *h* is the overall proportion of the population in the harvest. When reproductive values and proportion of quality classes in the harvest are considered an approximation of λ is obtained based on sensitivity analysis as:


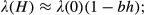


where *b* is the ratio of harvest proportion weighted by reproductive value of a quality class (*h*_rv_) and harvest proportion irrespective of reproductive value (*h*). Harvest proportion irrespective of reproductive value is:


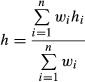


and harvest proportion weighted by reproductive value is:


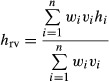


where *w*_*i*_ is proportion of population in age/quality class *i* at stable structure and *v*_*i*_ is the reproductive value for the *i*th age/quality class. The effects of harvest on λ when considering reproductive value will be smaller than the effects of uniform harvest irrespective of reproductive value when *b* < 1.0 (i.e., compensation occurs because harvest is proportionally higher in the population segments with lower reproductive value) and *b* can therefore be interpreted as the relative strength of harvest effects with stronger effects and less compensation as *b* approaches 1.0 (i.e., if *b* = 0 then harvest would hypothetically have no effect and if *b* = 1.0 no compensation would occur). We also examined the effects of harvest on overall population-level survival for the harvest scenario with the maximum effects of heterogeneity because survival is the parameter often examined to evaluate compensation.

## Results

### Multievent models

We included 31,167 individual female brant in our analysis and identified 2870 different capture histories. GOF tests provide strong evidence of heterogeneity. Following the results in Fletcher et al. (2012, pp. 208–209), components of TEST3.SR (*z* = 55.1) and TEST2.CT (*z* = −14.61), distributed as *N*(0, 1) under full homogeneity, indicated strong heterogeneity in capture probability, with, as expected a higher sensitivity of TEST3.SR. Other tests (nondirectional components of TEST3.SR and TEST2.CT + TEST3.SM and TEST2.CL, 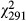 = 919.5, *P* = 0.000) indicated heterogeneity in survival probability. If all test components were considered, the variance inflation factor (*ĉ*) would equal 7.23 and 3.16 without the directional components directly linked to recapture heterogeneity. This latter value was used as an overdispersion factor in QAIC-based model selection because we considered only models that included heterogeneity in *P*. Because of the sensitivity of the GOF components used to estimate *ĉ* to heterogeneity in all parameters, we suspect that this *ĉ* estimate is still biased high (White 2002); however, we are unsure what lower value this estimate should be adjusted to and prefer instead to use a conservative approach to model selection (see also Lebreton et al. 2003).

A general model with full-year effects and heterogeneity had 338 identifiable parameters, QAIC = 41903.7, and served as a benchmark for model selection. Removal of heterogeneity from proportion of individuals in initial states, recruitment, and survival parameters, but leaving heterogeneity in recapture probability resulted in a model with the highest QAIC value (42557.301, for 123 identifiable parameters) of the 65 models considered, thus confirming the presence of strong demographic heterogeneity as observed from the goodness-of-fit tests, and not just heterogeneity in recapture probability.

We found strong support for removing year effects in recruitment and the interaction between year and age effects on survival probability (ΔQAIC = −275.8 for model without these effects). Year trending in survival probability was also not supported (ΔQAIC = +29.2 for model with trending). A model with a different, year-specific variation in HY and ASY survival, and no heterogeneity in ASY survival were preferable (ΔQAIC = −20.7) to a model with additive year effects for HY and ASY survival and heterogeneity across all age classes.

Age-specific variation in recruitment probability was not strongly supported as ΔQAIC = 0.5 between a model with no age-specific transition to breeding (105 parameters) and a model (107 parameters) with age-specific recruitment for age 2 and all other age classes (3+). Furthermore, the base model with age-specific recruitment for ages 2–5 had a ΔQAIC = +280.2 compared with the model without age-specific recruitment. Assuming a recruitment probability constant over age induces a cumulative recruitment curve slowly declining over years and ensures identifiability of the model. Models with full recruitment completed at age 5, 6, or 7 did not do better than these models with constant recruitment.

Models with additive effects of year and heterogeneity on capture probability generally had lower QAIC values than models with interactions between these factors as the top 17 models had additive effects. However, this result is somewhat in contrast to variability in components of TEST3.SR, which suggests a change in heterogeneity over time, the variability in the proportion of low-capturability individuals translating in an excess of individuals never seen again after first detection as a BR. For this same reason, we suspect that TEST3.SR components, which compare the proportion of individuals seen again or not seen again between newly marked and already marked individuals, may be sensitive to heterogeneity in initial-state probabilities rather than only to heterogeneity in recapture probability.

**Figure 1:**
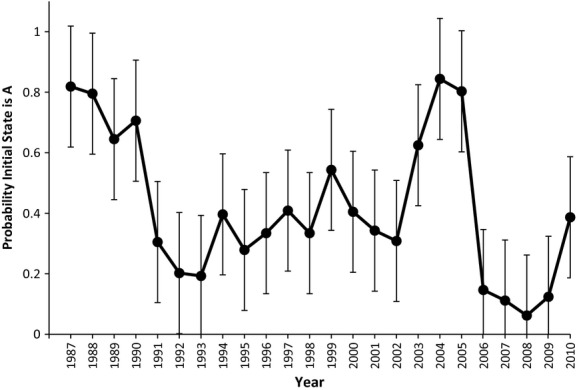
Year-specific probability that an individual was in state A at initial capture. Error bars are ±1 standard error.

**Figure 2:**
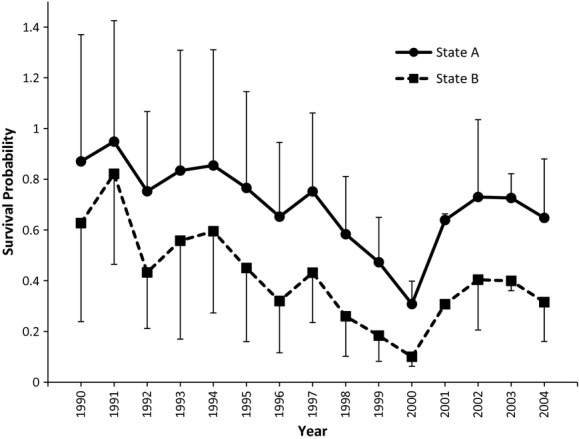
First-year survival probability of brant for states A and B. Errors bars are ±1 standard error in single direction for clarity.

The best approximating model included year-specific variation in probability that an individual was in group A or B at initial capture (Fig. 1) and was favored over the second (ΔQAIC = 3.7) and third best (ΔQAIC = 10.4) models, which included an additive relationship between proportion BR and PB and year or mass effects on HY survival, respectively. Therefore, we used estimates only from the top model. In this model, HY survival was modeled with an additive relationship between year and heterogeneity (Fig. 2) with differences in survival between high (A) and low (B) quality groups equal to 1.38 (SE = 0.84) on a logit scale or about 0.23 at average (0.50 low quality and 0.73 high quality) survival probability. ASY survival was constant and equaled 0.85 (SE = 0.004). Recruitment probability was constant across ages, but the rate of recruitment was higher for group A (0.54, SE = 0.214) than group B (0.31, SE = 0.028). Capture probabilities varied across years and included heterogeneity in an additive relationship. Capture probabilities were lower for group A than B and may indicate a sampling covariance that exaggerates heterogeneity in other parameters (see Discussion).

### Matrix models

Assuming 80% of the goslings produced were in the same quality class as the breeding female and equal clutch size between quality classes resulted in a projected λ = 1.058 (Table 1). Under these conditions, reproductive values of high-quality brant were about twice as high as the comparable states for low-quality brant. Differences in reproductive value increased considerably when we assumed the differences in quality also resulted in a difference in clutch size (5 vs. 3) and λ increased to 1.090 (Table 1). When we assumed no inheritance of quality (i.e., 50% of offspring in class) and equal clutch size, differences in reproductive values between high- and low-quality goslings and PBs were less than under the other conditions and equal among classes of BRs because of identical ASY survival in this class of birds (Table 1). High-quality BRs had the highest reproductive values and low-quality goslings the lowest reproductive values under every scenario.

**Table 1:** Population growth rates and reproductive values of brant for the six states under different scenarios of inheritance and heterogeneity

				Reproductive value					
				
Inheritance	Clutch size (high quality)	Clutch size (low quality)	λ	GOS-high	PB-high	B-high	GOS-low	PB-low	BR-low
0.8	4	4	1.058	0.174	0.251	0.292	0.046	0.097	0.140
0.5	4	4	1.044	0.135	0.192	0.222	0.074	0.156	0.222
0.8	5	3	1.090	0.234	0.346	0.408	0.002	0.004	0.006

GOS, gosling; PB, prebreeders; BR, breeders; high, high-quality individuals; low, low-quality individuals.

**Figure 3:**
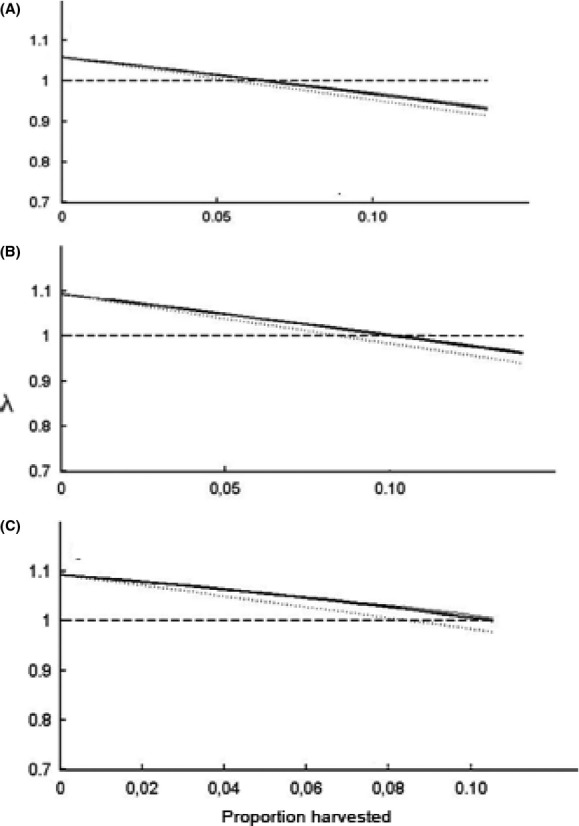
Population growth rate as a function of the proportion of the population harvested under scenario 1 (A), 2 (B), or 3 (C; Table 2). Thin dotted line = growth rate without considering heterogeneity (no compensation); thick solid line = growth rate with compensation for heterogeneity; thin solid line = growth rate with compensation for harvest parameter sensitivity.

**Figure 4:**
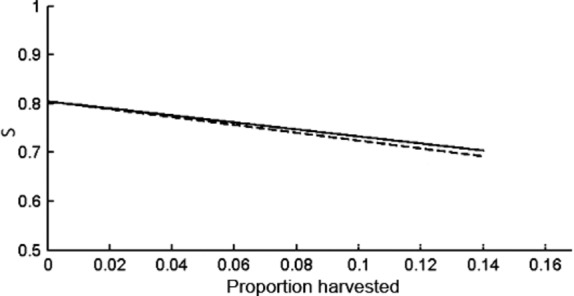
Changes in overall population survival (S) relative to changes in proportion of population harvested in a brant population without (dashed line) and with heterogeneity (solid line) as described under harvest scenario 3 in Table 2.

The compensation index (*b*) increased (compensation decreased), and the relative effects of harvest therefore increased when (1) differences in clutch size between classes decreased, (2) the proportion of high-quality individuals in the harvest increased, and (3) the proportion of BRs in the harvest increased. The compensation index was below 1.0 for all the scenarios we considered because the ratio of low-quality individuals in the harvest <0.51 (Table 2, Fig. 3). Under all scenarios and levels of compensation, λ was below 1.0 when overall harvest rate was >10%. Under scenario 3 and at 10% overall harvest, compensation through heterogeneity resulted in λ = 1.009; 0.028 higher (about 3% higher growth rate) compared with the situation when heterogeneity was not considered (λ = 0.981). We observed almost no response in population survival under the harvest scenario 3 because at stable structure, most of the population was composed of BRs, which exhibited no heterogeneity in survival (Fig. 4).

**Table 2:** Three scenarios of harvest for brant and resulting levels of compensation (*b*)

Scenario	Clutch size (high quality)	Clutch size (low quality)	Harvest ratio high/low	Harvest ratio pre-breeder/breeder	*b*
1	4	4	0.5	2	0.812
2	5	3	0.5	2	0.791
3	5	3	0.3	3	0.635

## Discussion

### Heterogeneity modeling

Our analysis provided strong evidence that heterogeneity in survival and recruitment was present in the brant population. To our knowledge, this is the first time that the levels of heterogeneity were quantified simultaneously for >1 parameter, and our approach may be useful for others examining heterogeneity. However, we acknowledge that additional advances would have improved our modeling. We did not simultaneously model heterogeneity in the full suite of population parameters (e.g., clutch size), and state space modeling (e.g., Borysiewicz et al. 2009) may be a useful tool for heterogeneity modeling when different types of data are available (e.g., longitudinal and survey data). Also, frailty models provided adequate structure for our questions, but modeling heterogeneity as a distribution may be preferable for other questions (Dorazio and Royle 2003). Finally, we could not resolve the link between estimates of capture probability and quality classes with higher-quality individuals having lower-capture probabilities. Collectively, we do not think these issues had much effect on our estimates, except to possibly inflate levels of heterogeneity in survival and recruitment; however, we believe our inferences are relevant to the population of interest.

### Sources of heterogeneity

We observed yearly variation in the proportion of individuals in the initial quality states. We suspect this heterogeneity is related to the quality of individuals breeding in a given year, the quality of the environment that goslings experienced prior to fledging, and genetic–environmental covariance (Sedinger and Chelgren 2007; Nicolai and Sedinger 2012). However, we are unclear why average mass of a cohort, which reflects quality of environment goslings experience prior to fledging, did not receive additional support in our analysis. Perhaps, the strength of the three causes of heterogeneity identified above varies through time, and a single description of these sources (e.g., gosling mass) is an inadequate description (Sedinger and Chelgren 2007).

Heterogeneity in annual survival of PBs and recruitment were consistent (additive for annual survival) and of similar magnitude; about 0.25 higher for high than low-quality individuals at mean values, perhaps reflecting similar sources of variation. Lower survival and recruitment of young brant led to proportionally fewer low-quality individuals alive and reproducing in the older age classes and therefore limited our ability to identify remaining heterogeneity in older age classes. However, past and more directed studies of survival and recruitment indicated that condition at fledging affects future survival and recruitment (Sedinger and Chelgren 2007; Sedinger et al. 2008). We did not attempt to model sources of variation in clutch size of BRs; however, the range of variation we considered is consistent with past studies (Nicolai and Sedinger 2012).

The effects of individual heterogeneity on population dynamics were tempered by how we parameterized heterogeneity in our models and also by our assumption about inheritance of quality. Although we did not observe heterogeneity in survival of adult brant, our results were sensitive to relatively small changes in the clutch size of adults because this class of individuals had the highest reproductive values. However, differences in reproductive values among, and within, classes of individuals were highly dependent on assumptions about inheritance of quality class, and these differences were minimized or eliminated when inheritance was removed. Based on genetic studies of related species (e.g., Lesser Snow Geese, *Chen caerulescens caerulescens,* Cooke et al. 1995) and our understanding of how brant goslings use brood-rearing areas (Lindberg and Sedinger 1998; Nicolai and Sedinger 2012), we suspect that quality is heritable either through direct genetic inheritance or through fidelity to high-quality environments or both. Therefore, harvest scenarios that included some level of inheritance of quality seem more appropriate than those that did not.

### Harvest dynamics of brant

We demonstrated that individual heterogeneity alone could explain the observed relationship between harvest and population dynamics (Johnson et al. 1984; Lebreton 2005). Under the scenarios we considered, compensation through heterogeneity resulted in about a 3% higher λ at the highest rates of harvest compared with an additive scenario. Although current rates of fall harvest for brant have declined to about 1% (Sedinger et al. 2007), total harvest (fall and spring) may still have effects on population dynamics because (1) density-dependent reductions in recruitment and abundance from degrading habitat (Sedinger and Nicolai 2011) have likely produced a population composed of proportionally more high-quality brant, (2) spring harvest has likely increased in recent years and has definitely become a larger component of the overall harvest, (3) spring harvest occurs mostly near breeding areas and likely includes mostly high-quality individuals because migration timing and routes of pre- and nonbreeders do not expose those groups to as much spring harvest (Sedinger and Nicolai 2011), and (4) in spring, a large part of low-quality individuals have been eliminated, due to the heterogeneity in first-year survival, and the differences in reproductive values between low- and high-quality individuals are smaller, being only the result of the difference in recruitment pace, and possibly, clutch size. Therefore, the potential for heterogeneity to compensate for harvest through differential removal of low-quality individuals is likely lower in the spring than the fall (Ward et al. 1997; Francis 2000; but see Sedinger and Nicolai 2011). However, we do not currently have adequate information on spring harvest rates or heterogeneity in reproduction of brant to effectively model effects of spring harvest.

### Heterogeneity and harvest dynamics

The concepts of individual heterogeneity and harvest are not new to hunting regulations. Populations are frequently managed for different quality individuals. For example, harvest regulations for large mammals commonly include restrictions on animals with different size antlers or horns, which may be linked to reproductive value of those individuals (e.g., Garel et al. 2007). Species with some phenotypic expression of quality (e.g., antler size) may more readily lend themselves to harvest regulations that account for individual heterogeneity. However, this type of phenotypic expression is more common for males than females, and in particular for polygynous populations. Harvest of males has less effect on population dynamics than that of females. Nonetheless, excessive harvest of males with some traits can lead to undesirable population effects (Coltman et al. 2003). Gender-specific regulations may therefore be one way to minimize the effects of harvest in the presence of individual heterogeneity in quality. A more general strategy is to target different quality classes when they are temporally or spatially segregated, and this may be an appropriate strategy for migratory bird harvest.

For migratory birds, most studies indicate that lower-quality individuals are more vulnerable to harvest (e.g., Greenwood et al. 1986; Dufour et al. 1993; Heitmeyer et al. 1993; Pace and Afton 1999) than are high-quality individual, but vulnerability is temporally and spatially variable. The potential compensating effects of heterogeneity and differential harvest likely decrease during the season as both the proportion of low-quality individuals in the population and differences in reproductive values between quality classes decline. Spatially, we think that the most likely reason for variation in vulnerability is related to timing of migration for low- and high-quality individuals and is rooted in the spatial segregation of low- and high-quality individuals at the end of the breeding season because of different molting strategies/regions (Hohman et al. 1992). The timing and pattern of migration may therefore differ because of variability in feather development, body condition, and local weather and habitat conditions. Low- and high-quality individuals may remain segregated during fall migration, and the extent and length of this segregation could vary annually. However, harvest regulations are generally based on calendar dates and in any given year harvest may impact individuals of different qualities, which may explain yearly variation in the effects of harvest on populations. The compensating effects of heterogeneity may be maximized by considering both the timing and location of harvest (Francis 2000; Conroy et al. 2005). For waterfowl specifically, we suggest that an individual heterogeneity model be explored as an alternative to the density-dependent models under consideration in current harvest management frameworks (e.g., Johnson et al. 1993).
